# Urodynamic evaluation of patients with localized prostate cancer before and 4 months after robotic radical prostatectomy

**DOI:** 10.1038/s41598-021-83143-x

**Published:** 2021-02-11

**Authors:** Dong Sup Lee, Seung-ju Lee

**Affiliations:** Department of Urology, St. Vincent’s Hospital, The Catholic University of Korea, College of Medicine, 93-6 Ji-dong Paldal-gu, Suwon, 16247 Korea

**Keywords:** Bladder, Prostate, Urology

## Abstract

Radical prostatectomy can alter the anatomy of the urinary bladder. We aimed to evaluate bladder function before and 4 months after radical prostatectomy using the urodynamic test and overactive bladder (OAB) symptom score. Among 70 prospectively enrolled patients, 61 patients completed the study. In the urodynamic test, bladder capacity and compliance did not change, the frequency of involuntary detrusor contraction decreased, the maximum flow rate and bladder outlet obstruction index improved, and the maximum urethral closure pressure (MUCP) deteriorated. Further evaluation of urodynamic parameters according to changes in symptoms was made. Although change in bladder compliance was correlated with changes in OAB symptoms, not the relative change of bladder compliance but the relative change in the MUCP was reliable factor when OAB symptoms were deteriorated. In general, prostatectomy did not deteriorate the condition of the detrusor; rather, change in the MUCP could be responsible for postprostatectomy OAB.

## Introduction

According to the Korean Statistical Information Service, 12,797 patients were newly diagnosed with prostate cancer in 2017, accounting for 10.5% of all new cases of cancer^1^. In the U.S., an estimated 174,650 patients were newly diagnosed with prostate cancer, accounting for 9.9% of all new cases of cancer^2^. When patients are diagnosed with localized prostate cancer, patients can choose the treatment modality, such as active surveillance, surgery, radiation therapy with or without androgen deprivation therapy, and androgen deprivation therapy alone, after careful discussion with their attending physicians. With the remarkable development of medical engineering and the need for operator convenience, the popularization of robotic surgery has been relatively rapid for prostatectomy compared to other types of surgery^3^. In regard to postprostatectomy incontinence, robotic radical prostatectomy seems to yield similar or better results compared to laparoscopic prostatectomy^4^. Furthermore, several surgical techniques have been introduced to overcome postprostatectomy urinary incontinence^5,6^. Nevertheless, Hoffman et al. reported that urinary incontinence was the most bothersome problem in patients who underwent surgery compared to other treatment options^7^.

In addition to urinary incontinence, issues regarding postprostatectomy overactive bladder (OAB) have been raised over the past 10 years^8^. The mechanism of symptomatic OAB after prostatectomy is still unclear. Matsukawa et al. suggested a urogenital mechanism in which the maximum urethral closure pressure (MUCP) plays a key role in generating OAB^9^. However, other possibilities, such as bladder outlet obstruction (BOO) or deterioration of the detrusor’s condition, including detrusor overactivity (DO), should be considered in the development of postprostatectomy OAB^10,11^.

**Figure 1:**
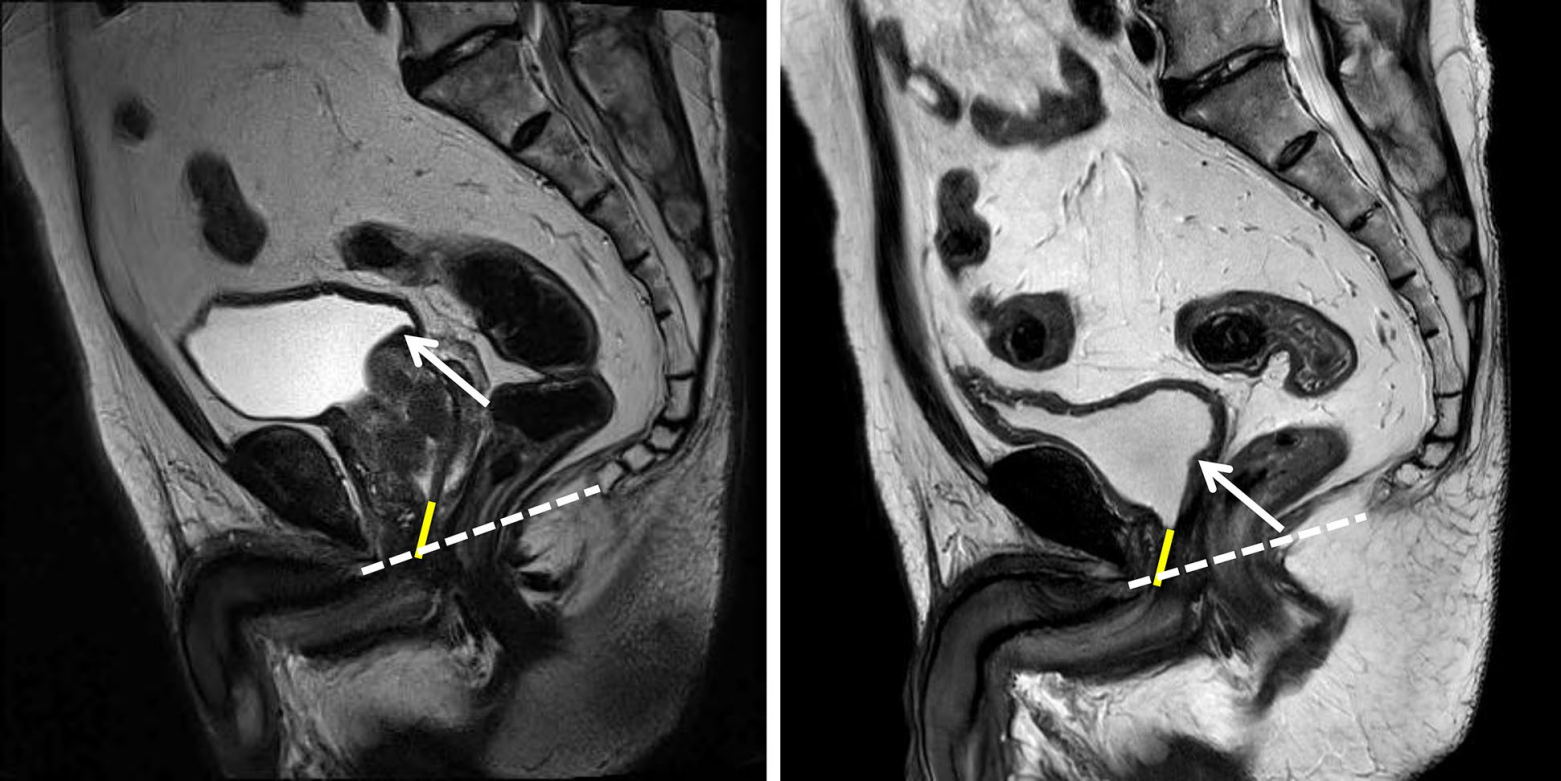
Anatomical changes in the urinary bladder after radical prostatectomy and the background for establishing the hypothesis of the present study. Left: Before prostatectomy, the level of the bladder neck was at the level of the upper margin of the symphysis pubis. Right: The membranous urethral length (yellow bar) was well preserved, but the levels of the urethral orifice (arrow) and de novo bladder neck moved downward (dotted line: pubococcygeal line). Before initiation of the present study, we had believed that postprostatectomy OAB could have occurred in response to the downwardly pulled urinary bladder showing deterioration of the storage phase-related urodynamic parameter.

We usually use magnetic resonance imaging of the prostate when prostate-specific antigen levels are elevated in patients who have undergone prostatectomy, which often shows that the lower portion of the urinary bladder is pulled downward without shortening of the functional urethral length (Fig. 1). It is reasonable to suspect that such an anatomical alteration that stretches the trigone area of the urinary bladder may deteriorate storage symptoms because (1) stretching the urinary bladder generates increased tension, which could affect bladder compliance^12^, and (2) the trigone area of the urinary bladder plays an important role in storage symptoms due to afferent C‐fibre-type trigonal nerves^13^. Therefore, we hypothesized that the urodynamic parameters of the storage phase, including bladder compliance and involuntary bladder contraction, would deteriorate after radical prostatectomy, which could contribute to OAB symptoms.

## Methods

### Ethics

The institutional review board in the St. Vincent's hospital approved the observational study design and access to the patients’ medical records (approval number: VC18TNSI0074, date of approval: April 24th, 2018). This study was performed in accordance with the Declaration of Helsinki. Written informed consent was obtained from all individual participants in the study.

### Patient selection and study design

**Figure 2:**
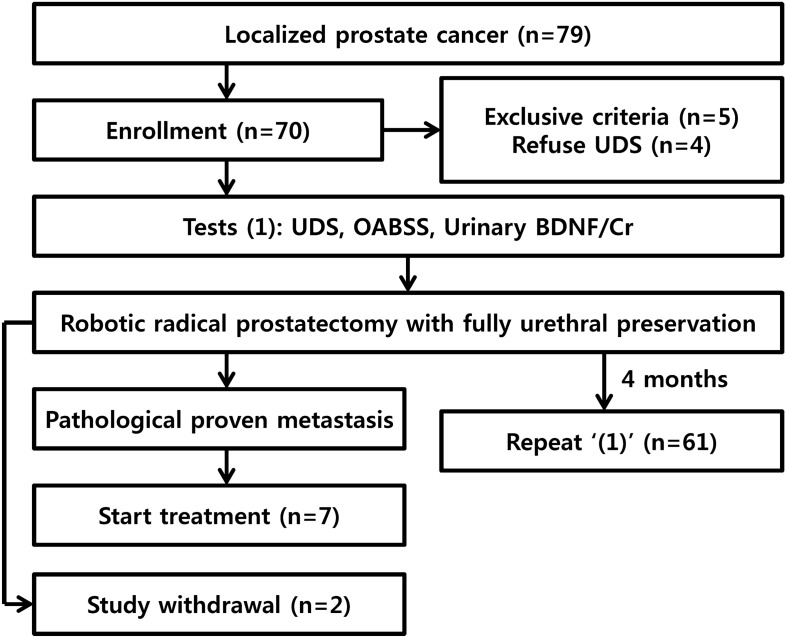
Study process. The OABSS questionnaire, urodynamic study and urinary BDNF/Cr evaluation were performed immediately after enrolment (1). These procedures were repeated 4 months after robotic radical prostatectomy (2). *UDS* urodynamic study, *OABSS* overactive bladder symptom score, *BDNF* brain-derived neurotrophic factor.

The present study is a prospective observational study involving consecutive patients diagnosed with localized prostate cancer from May 2018 to July 2019. A questionnaire evaluating the overactive bladder symptom score (OABSS) was provided to all patients. The patients were asked to collect urine samples for measurement of the urinary brain-derived neurotrophic factor to creatinine (BDNF/Cr) ratio. Finally, all patients underwent a urodynamic evaluation before prostatectomy. Four months after robotic radical prostatectomy, the aforementioned tests, including the questionnaire, urinary BDNF/Cr ratio measurement, and urodynamic evaluations, were repeated (Fig. 2).

The inclusion criteria were as follows: (1) age from 50 to 80 years; and (2) localized prostate cancer without metastasis (TNM stages should be T2–T3b N0 M0). With regard to extravesical causes of urinary symptoms, the exclusion criteria were as follows: (1) uncontrolled diabetes mellitus (HbA1c > 8.0)^14^; (2) a history of genitourinary tuberculosis; (3) neurological disease such as cerebral infarction or myelopathy^15^; and (4) psychotic disorders. When nodal metastasis was noted after robotic radical prostatectomy, the patient was excluded from the study, and relevant treatment was applied. Additional radiotherapy was seriously considered promptly after the study in patients with positive surgical margins, extraprostatic extension or seminal vesicle invasion without evidence of metastasis because radiotherapy could be applied 4–6 months after radical prostatectomy^16^, which is the reason why we set the end point at 4 months after surgery.

### Urodynamic test

The maximum flow rate (Qmax) was measured during noninvasive uroflowmetry. A Biocon 500 ultrasound scanner (Medline Industries, Inc., Mundelein, Illinois, USA) was used for postvoid residual urine volume (PVR) measurement after uroflowmetry. The urinary bladder pressure was measured using a 2-way 8-French catheter (Peters Surgical, Bobigny, France) at 40 mL/min of normal saline infusion and room temperature while the abdominal pressure was measured using a catheter with a PVC balloon placed in the rectum (Peters Surgical, Bobigny, France).

During the storage phase, bladder compliance was measured as the change in bladder volume divided by the change in bladder pressure. Stress urinary incontinence (SUI) was defined when urinary leakage was observed upon the Valsalva manoeuvre or coughing during cystometry. DO was documented when involuntary bladder contraction (IDC) was identified during cystometry. When patients felt the desire to void, a physician recorded this episode and allowed them to void. The detrusor pressure at the maximum flow rate (PdetQmax) was measured during the pressure flow study (voiding phase), and then the bladder outlet obstruction index (BOOI, PdetQmax—2xQmax) was calculated from the result^17^. The MUCP was measured during removal of the urethral catheter, which was inserted during the urodynamic study, and the value was defined as the maximum difference between the urethral pressure and the intravesical pressure, as previously described in the literature^18^.

### BDNF measurement

Urine samples were collected in 120-mL sterile tubes following a midstream capture protocol at 09:00–10:00 a.m. and promptly stored at – 80 °C. All reagents, including recombinant human BDNF, washing buffer, biotinylated antibody, streptavidin solution, TMB one-step substrate reagent, and stop solution, were included in the ELISA kit (MyBioSource, CA, USA), and the laboratory processes were performed according to the manufacturer’s protocol. Briefly, the process (manufacturer’s protocol) for BDNF measurement was as follows. First, all reagents and samples were brought to room temperature. Then, 100 μl of both the recombinant human BDNF and urine sample were mixed into each well and incubated in the plate for 150 min at room temperature under gentle shaking. After the solution was discarded, each well was washed with washing buffer (300 μl) using a multichannel pipette (Brand Tech Scientific, MD, USA) or autowasher (ELx405 Select Deep Well Washer, BioTek, VT, USA). Then, 100 μl of biotinylated antibody was applied to each well and incubated for 1 h at room temperature under gentle shaking. Subsequently, 100 μl of streptavidin solution was added to each well and allowed to stand for approximately 45 min, followed by the addition of 100 μl of TMB one-step substrate reagent to each well for 30 min of incubation in the dark under gentle shaking. Finally, 50 μl of stop solution was added to each well. Using an Infinite 200 PRO Series Multimode Reader (Tecan, Männedorf, Swiss), the mean absorbance was measured. We normalized the total urinary BDNF level to the concentration of urinary creatinine (BDNF/Cr level).

### Assessment of OAB symptoms

OAB is defined by subjective symptoms, including urinary urgency, with or without urgency incontinence and usually with urinary frequency, and nocturia, in the absence of infection or other obvious pathological features^19^. We employed a simple and widely used questionnaire, the OABSS questionnaire^20^, which incorporates OAB-wet and OAB-dry scores and scores OAB symptoms from 0 to 15.

### Procedure for robotic radical prostatectomy

The robotic radical prostatectomy procedure is briefly summarized as follows. With gentle caudal traction of the urinary bladder, the base of the prostate was dissected from the bladder neck. At that time, an incision was made between the most proximal part of the prostatic urethra and bladder neck. Then, the urethral catheter was withdrawn from the urinary bladder and immediately introduced though the incision window. An assistant surgeon (or the third arm) grasped the tip of the urethral catheter with laparoscopic alligator forceps and lifted it to facilitate posterior dissection of the prostate. The vas deferens and seminal vesicles were dissected, and then an assistant surgeon (or the third arm) grasped them and applied upward and lateral traction to facilitate pedicle dissection with or without a nerve-sparing procedure. Urethral dissection was performed with simultaneous downward traction of the prostate. Because a considerable part of the striated sphincter of the membranous urethra is located between the apex of the prostate and the colliculus seminalis^6^, careful dissection of the urethra from the apex of the prostate allows the colliculus seminalis to remain in the proximal part of the membranous urethra. Finally, the urethra was anastomosed with the bladder neck using 3–0 barbed running sutures (V-Loc, Covidien, Mansfield, MA).

### Statistics

First, to evaluate bladder function before and 4 months after prostatectomy, we set the target sample size as 54 using G*power version 3.1 with two tails, an effect size of 0.5, an α error of 0.5, and a 1-β error of 0.95. Concerning the patient drop-out rate (approximately 10–20%), we decided to enrol 70 patients with localized prostate cancer who wanted to undergo robotic radical prostatectomy. Missing values (preoperative pressure flow in one case and postoperative urodynamic parameters in one case) were omitted during the statistical evaluation. Statistical analyses were performed using the Statistical Package for the Social Sciences (SPSS version 20.0 for Windows; SPSS, Inc., Chicago, IL, USA). P-values < 0.05 were considered statistically significant.

## Results

In total, 70 of 79 patients were enrolled in the present study; four patients refused enrolment because of discomfort in the urodynamic evaluation, and 5 patients had exclusive conditions such as uncontrolled diabetes and/or a history of cerebral infarction. Among the 70 enrolled patients, two patients withdrew from the study, and 7 patients dropped out because of pathologically proven lymph node metastasis (Fig. 2). Therefore, 61 patients completed the follow-up evaluations; among the 61 patients, one patient failed to void during the preoperative pressure flow study, and a technical error in the postoperative urodynamic study occurred in one case. All patients were routinely discharged from the hospital on postoperative days 5 ~ 7 without perioperative complications. Tumour-related baseline patient characteristics are described in Table 1. We offered adjuvant radiotherapy to 16 patients promptly after completion of the present study, including 7 patients with T3a disease with margin positivity and 9 patients with T3b disease (regardless of margin positivity)^21^.

**Table 1:** Patients’ baseline characteristics (n = 61).

Age (year)	69.0 (61.0, 73.0)
Body mass index (kg/m^2^)	23.6 (21.9, 26.2)
Diabetes mellitus (%)	11 (18.0)
Hypertension (%)	29 (47.5)
Prostate size (mL)	29.4 (23.8, 40.0)
Prostate-specific antigen (ng/mL)	7.8 (5.3, 12.3)
**Gleason score (%)**	
Gleason score 6 or less	11 (18.0)
Gleason score 7 (3 + 4)	18 (29.5)
Gleason score 7 (4 + 3)	23 (37.7)
Gleason score 8 or more	9 (14.8)
**T stage (%)**	
T2	34 (55.7)
T3a	18 (29.5)
T3b	9 (14.8)
**Pathological details (%)**	
Transitional zone involvement	10 (16.4)
Ductal type	3 (4.9)
Tumour volume (> 5 mL)	15 (24.6)
Presence of PIN	37 (60.7)
Perineural invasion	46 (75.4)
Multiplicity	36 (59.0)
Nerve sparing procedure (%)	50 (82.0)
Surgical margin positive (%)	14 (22.9)
At apex (%)	5 (8.2)

Data are presented as the median with the interquartile range or the frequency (%).

OAB symptoms in the present cohort were relatively mild at the beginning of the study, and the mean symptom score was not different at the end of the study, while the OAB-dry (Q3) score decreased but the OAB-wet score (Q4) increased (Table 2). Regarding the urodynamic parameters, the Qmax and BOOI were significantly improved, whereas the MUCP deteriorated significantly. In contrast to our expectations, bladder compliance and the urinary BDNF/Cr ratio did not change, and the frequency of IDC decreased, which were the most important findings in the present study (Table 2).

**Table 2:** Comparison of the data before and 4 months after robotic radical prostatectomy (n = 61).

Parameters	Preop	Postop	Statistics
**OABSS**^**a**^			
Q1	0 (0, 1)	1 (0, 1)	0.284
Q2	2 (2, 3)	2 (1, 3)	0.330
Q3	1 (0, 3)	1 (0, 2)	0.026
Q4	0 (0, 1)	1 (1, 2)	0.007
Total	5 (3, 7.5)	4 (2, 8)	0.993
**Urodynamic parameters**^**b**^			
Qmax (mL/s)	7.8 (5.0, 10.0)	10.0 (7.1, 12.0)	0.001
Capacity (mL)	306.0 (248.0, 356.0)	287.5 (229.3, 340.8)	0.529
PVR (mL)	41.0 (17.0, 70.0)	22.5 (10.0, 56.0)	0.072
IDC (%)^c^	6 (9.8)	3 (5.0)	0.001
Compliance (ΔmL/Δpr)	57.0 (44.0, 80.0)	57.5 (41.0, 80.0)	0.664
BOOI	36.0 (28.5, 53.3)	28.5 (15.0, 40.0)	< 0.001
MUCP (cmH_2_O)	110.0 (90.0, 143.8)	81.5 (70.0, 105.0)	< 0.001
SUI		11 (18.3)	
Urinary BDNF/creatinine (ng/dL/ng/mL × 100)^b^	0.22 (0.15, 0.36)	0.21 (0.12, 0.28)	0.283
PSA (ng/mL)	7.83 (5.34, 12.27)	0.01 (0.01, 0.05)	< 0.001

Data are presented as the median with the interquartile range or the frequency (%).

*OABSS* overactive bladder symptom score, *Qmax* maximum flow rate, *PVR* postvoid residual urine volume, *IDC* involuntary detrusor contraction, *BOOI* bladder outlet obstruction index, *MUCP* maximum urethral closing pressure, *SUI* stress urinary incontinence, *BDNF* brain-derived neurotrophic factor, *PSA* prostate-specific antigen.

^a^Wilcoxon test, ^b^t-paired test, ^c^Fisher exact test.

Because only six patients showed preoperative IDC, we further evaluated bladder compliance in terms of detrusor function changes. Considering the relationship among changes in Q3, Q4 and urodynamic parameters in total cohort, the change in bladder compliance was significantly correlated with the changes in symptoms (Supplementary Table 1), however, considering whether OAB symptoms were deteriorated or not, the MUCP alone showed significant relative changes (Table 3 and 4). The relative change in bladder compliance remained as a significant factor when we considered the resolution of OAB symptoms.

**Table 3:** Relative change in urodynamic parameters according to change in Q3 (n = 60).

Q3 (urgency)	Improved or unchanged (n = 44)	Deteriorated (n = 16)	Statistics
Compliance	0.00 (− 0.07, 0.29)	− 0.04 (− 0.33, 0.13)	0.095
Qmax	0.33 (0.00, 0.67)	0.32 (− 0.21, 0.95)	0.314
Capacity	− 0.02 (− 0.21, 0.19)	− 0.10 (− 0.21, 0.16)	0.969
PVR	− 0.27 (− 0.75, 0.39)	− 0.33 (− 0.61, 0.21)	0.596
BOOI	− 0.44 (− 0.62, 0.0)	− 0.33 (− 0.48, 0.29)	0.784
MUCP	− 0.14 (− 0.40, 0.06)	− 0.31 (− 0.41, − 0.18)	0.010
BDNF/Cr	− 0.25 (− 0.59, 0.40)	0.19 (− 0.10, 1.27)	0.094

Q3 (urgency)	Deteriorated or unchanged (n = 33)	Improved (n = 27)	Statistics
Compliance	− 0.05 (− 0.26, 0.09)	0.13 (− 0.03, 0.34)	0.024
Qmax	0.33 (− 0.26, 0.67)	0.29 (0.00, 0.74)	0.629
Capacity	− 0.02 (− 0.20, 0.19)	− 0.02(− 0.24, 0.15)	0.969
PVR	− 0.33 (− 0.67, 0.36)	− 0.21 (− 0.78, 0.19)	0.238
BOOI	− 0.09 (− 0.58, 0.24)	− 0.47 (− 0.63, 0.00)	0.156
MUCP	− 0.25 (− 0.42, 0.08)	− 0.18 (− 0.38, 0.16)	0.170
BDNF/Cr	0.19 (− 0.09, 0.73)	− 0.44 (− 0.67, 0.05)	0.027

The relative change in each urodynamic parameter was calculated ((postoperative value – preoperative value)/preoperative value). Data are presented as the median with the interquartile range.

Each statistical value was calculated by Student’s t-test.

*Qmax* maximum flow rate (mL/s), *PVR* postvoid residual urine volume (mL), *BOOI* bladder outlet obstruction index, *MUCP* maximum urethral closing pressure (cmH_2_O).

**Table 4:** Relative change in urodynamic parameters according to change in Q4 (n = 60).

Q4 (UUI)	Improved or Unchanged (n = 36)	Deteriorated (n = 24)	Statistics
Compliance	0.00 (− 0.07, 0.22)	− 0.04 (− 0.21, 0.19)	0.209
Qmax	0.25 (0.00, 0.67)	0.37 (− 0.23, 0.94)	0.173
Capacity	− 0.02 (− 0.21, 0.15)	− 0.08 (− 0.22, 0.33)	0.653
PVR	− 0.23 (− 0.66, 0.28)	− 0.33 (− 0.73, 0.38)	0.561
BOOI	− 0.37 (− 0.59, 0.17)	− 0.24 (− 0.61, 0.0)	0.633
MUCP	− 0.13 (− 0.38, 0.06)	− 0.35 (− 0.44, − 0.13)	0.028
BDNF/Cr	− 0.33 (− 0.63, 0.37)	0.07 (− 0.23, 0.95)	0.043

Q4 (UUI)	Deteriorated or unchanged (n = 51)	Improved (n = 9)	Statistics
Compliance	0.00 (− 0.20, 0.16)	0.20 (0.05, 0.76)	0.040
Qmax	0.32 (− 0.19, 0.74)	0.40 (− 0.05, 0.67)	0.713
Capacity	− 0.02 (− 0.23, 0.24)	− 0.03 (− 0.10, 0.13)	0.444
PVR	− 0.33 (− 0.74, 0.31)	0.10 (− 0.25, 0.32)	0.978
BOOI	− 0.34, (− 0.64, 0.16)	− 0.38 (− 0.53, 0.00)	0.536
MUCP	− 0.26 (− 0.41, − 0.04)	− 0.08 (− 0.33, 0.17)	0.264
BDNF/Cr	0.06 (− 0.42, 0.66)	− 0.55 (− 0.70, − 0.36)	0.068

The relative change in each urodynamic parameter was calculated ((postoperative value – preoperative value)/preoperative value). Data are presented as the median with the interquartile range.

Each statistical value was calculated by Student’s t-test.

*UUI* urgency urinary incontinence, *Qmax* maximum flow rate (mL/s), *PVR* postvoid residual urine volume (mL), *BOOI* bladder outlet obstruction index, *MUCP* maximum urethral closing pressure (cmH_2_O).

## Discussion

We hypothesized that urodynamic parameters of the storage phase, including bladder compliance and IDC, would deteriorate after radical prostatectomy because the lower portion of the urinary bladder should be pulled downward to some degree after prostatectomy. DO (IDC) may be one of the major urodynamic parameters for evaluation when assessing OAB symptoms. A report showed that 19% of men aged over 60 years have OAB symptoms^22^. However, Digesu et al. conducted a study where only 18.7% of patients with OAB showed DO^23^. Therefore, statistically, the prevalence of IDC might have been expected to be low especially when the patients were enrolled regardless of OAB symptoms. Therefore, we determined the minimum sample size (n = 54) to compare preoperative and postoperative bladder compliance (see the statistics in the Methods section).

In contrast to our hypothesis, bladder compliance and OABSS did not change in this cohort, and the frequency of IDC even decreased. Furthermore, the urodynamic parameters reflecting BOO, such as the Qmax, PVR and BOOI, were improved 4 months after prostatectomy. The only parameter that deteriorated was the MUCP (Table 2). Therefore, in general, we can conclude that the anatomical changes in the urinary bladder after robotic radical prostatectomy do not deteriorate the detrusor’s condition. In this point, it would be interesting to investigate urodynamic parameters among which exerted an influence on OAB symptoms changes. In general, symptoms (Q3 and Q4) changes were significantly associated with the change in bladder compliance rather than the changes in BOOI and/or MUCP (Supplementary Table 1). However, the MUCP alone was a risk factor for deterioration of those symptoms changes, whereas the bladder compliance could be a factor in cases of OAB resolution (Tables 3 and 4). Therefore, we could expect improvement of bladder compliance and subsequent OAB symptom resolution under the condition of relatively preserved MUCP. Therefore, the change in MUCP would be crucial on the development of postprostatectomy OAB and the resolution of OAB.

The present outcomes are supported by a recent study conducted by Matsukawa et al. in which the authors concluded that the postoperative MUCP was the most related factor to de novo OAB^9^. Though the authors of the previous study suggested a novel mechanism of postprostatectomy OAB, they included patients who had urgency scale (Q3) scores of 2 or more, which has been suggested as a cut-off value for the diagnosis of OAB in clinical research using the OABSS questionnaire^20^. However, the mechanism of postprostatectomy OAB is complex and has not yet been confirmed. There might be a contradiction when authors suggest change in urethral function (MUCP) as a risk factor for postprostatectomy OAB while simultaneously using only the Q3 scale which might be affected by storage-phase detrusor function. With the manner, they could not explain patients complaining of deterioration of urgency urinary incontinence without deterioration of urgency; could not explain patients complaining of urgency urinary incontinence with an urgency scale score of ‘1’. Therefore, to determine de novo OAB in patients treated with prostatectomy, physicians should not confine the diagnostic paradigm to the specific urgency scale (e.g. Q3 score 2 or more). In the present study, based on our results (Tables 3 and 4, and Supplementary Table 1), we think that patients with de novo OAB experience urgency because they might dribble when the bladder is sufficiently full and thus feel a voiding desire.

The MUCP has been reported to decrease as much as 50 cmH_2_O immediately after robotic radical prostatectomy^24^, and our results showed that the mean MUCP was 24 cmH_2_O lower than the baseline value at 4 months after radical prostatectomy. Even minimal narrowing of the urethra has been found to increase the internal resistance to flow by the fifth power of the change in radius^25^. Therefore, we can suggest evaluation of the MUCP for empirical management of postprostatectomy OAB with or without management of the detrusor’s condition with drugs such as antimuscarinics and/or beta-3 agonists in the absence of BOO.

Several methods have been suggested to improve the postoperative MUCP^26^. Pelvic floor muscle training is a classical method to enhance the MUCP^27^. Tienforti et al. found that perioperative pelvic floor muscle training not only could improve urinary incontinence but was also effective for decreasing OAB symptoms at 3 and 6 months after radical prostatectomy^28^, which can be explained by the relationship between the MUCP and OAB.

The resolution of OAB symptoms in association with improvements in bladder compliance and/or IDC may be due to emancipation of the urinary bladder from BOO, as bladder compliance is known to be improved by relieving BOO^29^. In the present study, changes in OAB symptoms was associated with changes in bladder compliance; however, we could not find any association between detrusor function changes and change in the BOOI (*p* = 0.489, *Pearson’s correlation coefficient* = − 0.092), which we attribute to the fact that (1) the BOOI improved in most cases, (2) the median value of BOOI was 36, and (3) the study period was relatively short (only 4 months) for assessing change in detrusor function.

The role of the detrusor’s condition in the changes in OAB symptoms was supported by the urinary BDNF/Cr level. BDNF has been known as a regulator of urinary bladder function and is produced in the bladder by urothelial and smooth muscle cells upon stretching to sensitize underlying bladder afferent C fibres^30^. If the altered anatomy of the lower portion of the urinary bladder can influence urinary function in terms of the ‘stretching effect’ or if other factors after prostatectomy contributed to the elevation of intravesical pressure, researchers may assume that BDNF expression may increase after prostatectomy. Recently, researchers have used neurotrophins, such as NGF or BDNF, to investigate storage-phase bladder function. Liu et al. compared OAB symptoms according to NGF levels and found that the NGF level was increased in patients with BOO and OAB symptoms and decreased after medical treatment^31^. Sekerci et al. investigated the value of urinary BDNF in assessing the response to botulinum toxin in patients with neurogenic DO^32^. The authors identified that the BDNF level was correlated with bladder compliance. Their findings strengthened our results describing storage-phase bladder conditions, such as bladder compliance.

The limitations of the present study should be mentioned. The main limitation was the small sample size. In addition, we could not definitively exclude postoperative bladder neck contracture because the study design did not include routine postoperative cystoscopy. Furthermore, we used a single questionnaire (OABSS) for symptom assessment; with a bladder diary, a more detailed evaluation for the number of urgency episodes, urinary incontinence, and frequency could have been completed.

In conclusion, robotic radical prostatectomy did not deteriorate the detrusor’s condition, including compliance and/or IDC. In cases of OAB symptom deterioration, the MUCP may exert a central role among urodynamic parameters.

## Supplementary Information

Supplementary Table S1.

## Data Availability

Data from the present study will be available at https://osf.io/kcwst/.
