# Supplementary material for: Urodynamic evaluation of patients with localized prostate cancer before and 4 months after robotic radical prostatectomy
Source: Sci Rep. 2021 Feb 11;11:3632. doi: 10.1038/s41598-021-83143-x (PMC7878735; doi:10.1038/s41598-021-83143-x)
Supplement: Supplementary file 1 — Supplementary Table S1. [file 41598_2021_83143_MOESM1_ESM.docx]

Supplementary Table 1. The relationship of the changes in OAB symptoms with the changes in urodynamic parameters

| Q3 (urgency) | p-value | *B* | 95% CI for *B* |
| --- | --- | --- | --- |
| Compliance | 0.003 | -0.037 | -0.062~-0.013 |
| BOOI | 0.240 | 0.009 | -0.006~0.025 |
| MUCP | 0.348 | -0.006 | -0.019~0.007 |
| Q4 (UUI) | p-value | *B* | 95% CI for *B* |
| Compliance | 0.099 | -0.017 | -0.038~0.003 |
| BOOI | 0.754 | 0.002 | -0.011~0.016 |
| MUCP | 0.235 | -0.007 | -0.017~0.004 |

Instead of the relative change ((post-pre)/pre), the difference (post-pre) between preoperative and postoperative values was applied for each parameter because denominators in some of OABSS scores were ‘0’.

Data were calculated using multivariate linear regression analysis.

OAB: overactive bladder symptom, UUI: urgency urinary incontinence, BOOI: bladder outlet obstruction index, MUCP: maximum urethral closing pressure, *B*: Unstandardized coefficient
